# A Randomized Controlled Trial Testing the Effectiveness of Coping with Cancer in the Kitchen, a Nutrition Education Program for Cancer Survivors

**DOI:** 10.3390/nu12103144

**Published:** 2020-10-15

**Authors:** Melissa Farmer Miller, Zhongyu Li, Melissa Habedank

**Affiliations:** 1 Department of Public Health, College of Health Sciences, Arcadia University, Glenside, PA 19038, USA; millerm@arcadia.edu; 2 Johns Hopkins Bloomberg School of Public Health, Baltimore, MD 21205, USA; lilizy597@gmail.com; 3 American Institute for Cancer Research, Arlington, VA 22209, USA

**Keywords:** cancer survivors, health behavior intervention, diet and nutrition

## Abstract

Following a diet rich in whole grains, vegetables, fruit, and beans may reduce cancer incidence and mortality. The aim of this study was to investigate the effect of Coping with Cancer in the Kitchen (CCK), an 8 week in-person program offering education, culinary demonstrations and food tasting, and psychosocial group support, compared to receiving CCK printed materials by mail on knowledge, confidence, and skills in implementing a plant-based diet. A total of 54 adult cancer survivors were randomly assigned to intervention (*n* = 26) and control groups (*n* = 27) with assessments at baseline, 9, and 15 weeks via self-administered survey. The response rate was 91% at 9 weeks and 58% at 15 weeks. The majority of our study participants were female breast cancer survivors (58%) who had overweight or obesity (65%). Compared with the control, there were significant (*p* < 0.05) increases in intervention participants’ knowledge about a plant-based diet at weeks 9 and 15, reductions in perceived barriers to eating more fruits and vegetables at week 9, and enhanced confidence and skills in preparing a plant-based diet at week 15. There was a significant reduction in processed meat intake but changes in other food groups and psychosocial measures were modest. Participation in CCK in person increased knowledge, skills, and confidence and reduced barriers to adopting a plant-based diet. Positive trends in intake of plant-based foods and quality of life warrant further investigation in larger-scale studies and diverse populations.

## 1. Introduction

The overall aging of the United States population and changing prevalence of risk factors, including obesity, have increased the incidence of many types of cancer while advances in the early detection and treatment of cancer have led to reduced cancer mortality. These factors have combined to dramatically increase the number of cancer survivors [1,2]. Nearly 17 million people in the US were living with a history of a cancer diagnosis as of January 2019. This number is projected to grow to more than 22 million by 2030 and to more than 26 million by 2040 [1,3].

The importance of diet for cancer survivors is indicated by the accumulating evidence that a healthier diet after a cancer diagnosis can lead to improved treatment response, recovery, side-effect management, and disease outcomes [4,5,6,7,8]. The World Cancer Research Fund/American Institute for Cancer Research’s (AICR’s) Third Expert Report, *Diet, Nutrition, Physical Activity and Cancer: a Global Perspective*, states ten Cancer Prevention Recommendations [9]. Six of these recommendations focus on aspects of diet, including following a dietary pattern rich in whole grains, vegetables, fruit and beans, and limiting consumption of red meat and processed food, to reduce cancer incidence and mortality. A substantial body of research has demonstrated the benefits of adherence to the AICR’s Cancer Prevention Recommendations, including a lower risk of cancer incidence, recurrence, and death [10,11,12,13,14].

Despite the growing evidence supporting positive changes in diet to prevent cancer-related morbidity and mortality, most cancer survivors’ adherence to the AICR’s dietary recommendations is low. A recent systematic review and meta-analysis concluded that only 34% of cancer survivors met the recommendations for fruit and vegetable (F&V) intake and only 31% and 47% for fiber and red meat intake, respectively, while 87% of cancer survivors met the recommendations for smoking and 83% for alcohol intake [15], which suggests potential opportunities for interventions to improve the dietary patterns and decisions among cancer survivors. Diet/nutrition was recognized as a common concern in an observational survey research study of cancer patients and survivors in the community across the US [16] and among members of a community-based cancer care organization [17]. Further, cancer survivors frequently experience low quality of life partly attributed to nutritional problems and thus report high demand for nutritional support [18,19]. In fact, diagnoses of cancer often motivate individuals to make lifestyle changes [20,21,22]. Nonetheless, cancer survivors may find it difficult to act on their intentions and can struggle to achieve their goals when they are not given necessary tools such as specific action plans and evidence-based information for making and sustaining behavioral changes [20,23,24]. 

Coping with Cancer in the Kitchen (CCK) was initiated to help fill this gap between lifestyle recommendations and the sustained adoption of improved lifestyle behaviors among post-diagnosis and post-treatment cancer survivors. CCK is an 8 week in-person program offering multidisciplinary support for cancer survivors that includes nutrition education, culinary demonstrations and food tasting. It also offers facilitated group discussions with structured goal setting to address psychosocial health, and it introduces simple and effective techniques for coping with cancer-related stress. The first phase of research was conducted in 2017 to test the feasibility and acceptability and to determine the preliminary effect sizes of the CCK program. These promising results were used to inform refinements in both the CCK curriculum and the training [25]. In 2019, the next phase of research was conducted as a randomized controlled trial to rigorously evaluate the efficacy of the in-person CCK program versus receiving printed CCK materials. The objectives of this paper are to describe the randomized controlled trial, report the results, and examine whether the in-person CCK program (the intervention group) increased knowledge, skills, and confidence in adopting a plant-based diet as well as made positive changes in dietary intake and quality of life compared to the delivery of the CCK program through printed materials (the control group).

## 2. Materials and Methods 

### 2.1. Program Overview 

CCK was developed according to evidence-based concepts, including AICR’s Cancer Prevention Recommendations, AICR’s Foods that Fight Cancer™, and AICR’s New American Plate^®^. CCK is a response to the needs of cancer survivors, registered dietitians, and other health professionals who lamented the lack of an evidence-based, standardized curriculum specifically for cancer survivors. A core principle guiding the design and development of CCK was to ensure that it benefitted communities in which it would be implemented. To achieve this goal, the design and development of CCK’s evidence-based curriculum involved multiple partners across its first and second phases of research, including AICR, Living Plate (Far Hills, NJ, USA), Cancer Support Community (CSC; Washington, DC, USA), CSC of Central New Jersey (Bedminster, NJ, USA), registered dietitians and mental health practitioners. The CCK intervention was primarily guided by the Social Cognitive Theory (i.e., knowledge and skills development, self-efficacy, and observational learning mediate behavior change) [26] and the Transtheoretical Model (i.e., stages of change) [27,28]. Its psychosocial components were guided by CSC’s affiliate model, which empowers individuals impacted by cancer to improve their health and well-being through active participation in community-based programs and active engagement with their health care team [29,30]. Furthermore, it is indicated that enhanced learning occurs in a small group defined by a shared cancer experience with professional and peer support to motivate behavior change [31,32]. 

Often the cost of food can be a barrier to trying new foods and purchasing perishable food items, both of which are important to adopting a plant-based diet. With this in mind, the CCK program’s recipes were designed to include relatively basic, whole foods that can be found at standard grocery stores; the serving sizes are small to limit the quantity of ingredients needed and the potential for spoilage; and no name brands are explicitly recommended. As well, a variety of recipes at various price points are provided so that facilitators can choose to demonstrate the recipes that are most appropriate for their participants and communities, and the curriculum includes time for the facilitators and participants to discuss possible recipe variations and ingredient substitutions. Finally, each week, the facilitators probe for perceived barriers to adopting a plant-based diet related to each module topic (e.g., veggies, snacks, whole grains, and breakfasts) and addresses financial concerns.

The first CCK pilot study assessed the feasibility, acceptability, and pre–post-impact of the program by a single-arm intervention among 21 adult cancer survivors in 2017 [25]. Participants reported increased confidence preparing a variety of plant-based foods (*p* = 0.002), perceived control over cancer (*p* = 0.034), perception of dietary quality (*p* = 0.009), and weekly behavioral capability, including food and nutrition knowledge (*p* < 0.001). There was a non-significant (NS) trend towards increased F&V and whole grain intake with moderate effect sizes (0.2–0.5) for intake of beans and legumes, vegetables, and cooked whole grains like brown rice and quinoa. This single-arm pilot study achieved enrollment of 88% of the accrual target; program attendance at each session ranged from 48% to 100%. Participant satisfaction was positive with 100% of participants very satisfied (9 or 10 on 11-point Likert scale) with the cooking demonstrations, 93% very satisfied with the facilitated group discussions, and 87% very satisfied with the nutrition education.

### 2.2. Study Design 

We conducted a two-arm, randomized controlled trial; cancer survivors were randomized to receive the 8-week CCK in-person multidisciplinary program immediately (intervention arm) or to receive CCK printed materials (control arm). Data was collected through self-administered patient surveys completed at baseline, post-intervention, and follow-up. A maximum total sample size of 60 (30 in each group) with 10% loss to follow-up and type I error of 5% provides >80% power to detect a difference (effect size) of at least 0.8 standard deviations between intervention and control groups (two-sample means test). This study was conducted according to the guidelines laid down in the Declaration of Helsinki, and all procedures involving research study participants were approved by Ethical and Independent Review Services (E&I) Institutional Review Board (Lee’s Summit, MO, USA; Approval code: 19048-01). Written informed consent was obtained from all subjects/patients during enrollment. The trial was retrospectively registered with ClinicalTrials.gov (Identifier: NCT04528615).

### 2.3. Study Population

Cancer survivors were recruited from community members served by CSC-Los Angeles (LA, CA, USA) and Fanwood-Scotch Plains YMCA (Scotch Plains, NJ, USA) from April to June 2019, largely by social media, emailed letters of invitation to affiliate members, community-based presentations at cancer support groups, and fliers. The research sites in Los Angeles, CA, and Scotch Plains, NJ, were selected to represent a diversity of cancer survivors—from the west and east coasts of the US, respectively; from urban and small township/suburban regions, respectively; and from sites that provide programming specific to cancer or with a general community-based focus, respectively. Eligibility criteria included: (1) 18 years of age or older; (2) ever been diagnosed with cancer; (3) able to attend at least seven of the eight total sessions with mandatory first and last sessions; (4) willingness to be randomized and adhere to study protocol; (5) completed active cancer treatment (not including hormonal or other similar agents, e.g., tamoxifen). If potential candidates had not completed active cancer treatment, research staff determined eligibility if side effects of the current treatment had not affected sense of taste causing difficulty eating healthy foods, like F&V or whole grains, and had not caused a level of fatigue that would impede ability to attend an 8-week program, shop for healthy foods, and prepare recipes.

### 2.4. Randomization

Consenting participants were randomly assigned to 1 of 2 treatment groups: CCK intervention or printed materials control. A unique computer-generated list for each of the two research sites randomly sequenced intervention and control assignments. Allocation to the intervention or control groups was concealed until all participants had been enrolled at a site. Once the final participant was enrolled, research staff broke the treatment code and assigned participants in order of enrollment to either the intervention group or the control group by adding them to the randomized list of intervention and control assignments. The local research staff notified participants in the intervention group of the start date for the CCK program. Participants in the control group were notified of their group assignment and mailed a baseline survey with a postage paid return envelope approximately one week before the start of the in-person CCK program with instructions to complete the survey within two weeks.

### 2.5. CCK Onsite Teams

CCK was facilitated by a multidisciplinary team. Registered dietitians were trained to educate participants about AICR’s Cancer Prevention Recommendations and two evidence-based programs: AICR’s New American Plate^®^ and AICR’s Foods That Fight Cancer™. Licensed social workers were trained to facilitate group support and discussions that strategized how participants could overcome psychosocial barriers to nutrition behavior change in the context of cancer survivorship, equip them with strategies to help with cancer-related stress, and encourage goal setting. In addition, the registered dietitian or a culinary assistant demonstrated convenient, easy, and tasty ways to prepare and cook plant-based foods and offered tastings. Prior to facilitating CCK, facilitators completed a required two-part live virtual training about how to implement the CCK program with intervention fidelity, and they met weekly by phone with researchers using a semi-structured moderator guide to address any questions or problems that arose during program implementation. An evaluation of the training program indicated a high level of knowledge about CCK research procedures and preparedness to deliver program content.

### 2.6. CCK Intervention Group

Components of the CCK intervention are summarized in Table 1. CCK participants attended eight, in-person, 90-min classes convened weekly at their community-based organizational facility. The schedule at each site included a 1-week midterm break for a national holiday and staff travel, so the complete program extended to 9 weeks. Weekly themes included beans and whole grains, one-pot meals, breakfast and snacks, comfort foods, veggies, and building a Foods that Fight Cancer kitchen. To encourage attendance, some participants were sent email reminders before some classes and some absentees were contacted by phone.

### 2.7. Printed Materials Control Group

The control group received printed CCK educational materials including 7 written summaries of weekly nutrition content and 14 recipes that emphasized the weekly nutrition themes (see Table 1). (Only 7 written summaries were provided because the eighth session was a reflection and review session with no new nutrition information introduced.) Research staff mailed the materials to control participants upon completion of the baseline survey. The control group was not contacted again except for follow-up surveys at 9 and 15 weeks. Upon completion of the 9-week survey, control participants were emailed a $10 gift card.

### 2.8. Data Collection/Participant Survey

All data was collected through self-administered participant survey at baseline (pre-test, 0 week), post-intervention (9 week), and at follow-up (15 week). For participants in the CCK intervention group, the baseline and post-intervention surveys were completed in person at the beginning of the first and last CCK classes, respectively. The baseline and post-intervention surveys (with a postage-paid return envelope) were mailed to the control participants at their home within approximately one week of in-person CCK program commencement and conclusion. The 15-week online follow-up survey was completed by participants in both the intervention and control groups. A link to the online survey was emailed 6 weeks from the last session, or 15 weeks from baseline, with a window to complete the follow-up survey of 15 to 18 weeks. Participants were contacted by email or phone as a reminder to complete the questionnaires, as needed.

### 2.9. Baseline and Outcome Measures

The primary outcomes included knowledge about a plant-based diet (average score of 6 items; Cronbach’s alpha = 0.80), confidence preparing a variety of plant-based foods (average score of 14 items; Cronbach’s alpha = 0.75), and skills to practice a plant-based diet (average score of 5 items; Cronbach’s alpha = 0.88) (see Table 1).

Secondary outcomes were measured using validated instruments for dietary intake (National Cancer Institute Dietary Screener Questionnaire) [35]; general quality of life (FACT-G7) [37]; psychological distress (PHQ-4) [38]; fatigue (single item from the Fatigue Symptom Inventory [39]); and emotional support (SOC8) [40]. We modified an existing scale [33,34] to measure perceived barriers to eating more F&V (average score of 15 items; Cronbach’s alpha = 0.89) and whole grains (average score of 14 items; Cronbach’s alpha = 0.83). ‘Perceived barriers’ was originally conceptualized as a moderating variable (those with fewer perceived barriers might experience a greater benefit from the CCK intervention) rather than an outcome variable and, for that reason, was not measured at 15 weeks follow-up. However, the findings indicate a reduction in barriers in the CCK intervention group, so we included ‘perceived barriers’ as an outcome.

Additional survey items included sociodemographic characteristics, disease characteristics, and health status.

### 2.10. Statistical Analysis

Descriptive statistics were calculated overall and by study group. Means and standard deviations are presented for continuous variables and frequencies and percentages are presented for categorical variables. We assessed the comparability between study groups using two-sample *t*-tests for continuous variables and Fisher’s exact test for categorical variables and pre–post differences within study groups using paired *t*-tests. We used multiple regression analysis to estimate the difference between the CCK intervention and control groups at 9 weeks (post-intervention) and at 15 weeks (follow-up) adjusting for baseline (pre-test) levels of the dependent variable and research site (stratification variable). We considered a *p*-value < 0.05 statistically significant.

Effect-size calculations were also used as a standard for determining a meaningful treatment effect using Cohen’s criteria for small, medium, and large effect sizes of 0.2, 0.5, and 0.8, respectively [41]. Standardizing the observed changes by the standard deviation (SD) allows for the comparison of the effect size magnitude across outcomes and can provide a meaningful reference for the future evaluation of the program in its implementation and dissemination. We calculated the *ES* statistic for the effect size, a form of Cohen’s effect size index, as the mean of the changes in outcome scores for each study group at baseline and post-intervention (9 week) divided by the baseline SD [42]. Thus,

(1) $$ES_{group}=\frac{{\bar{x}}_{Time2}-{\bar{x}}_{Time1}}{SD_{Time1}}$$

## 3. Results

**Figure 1:**
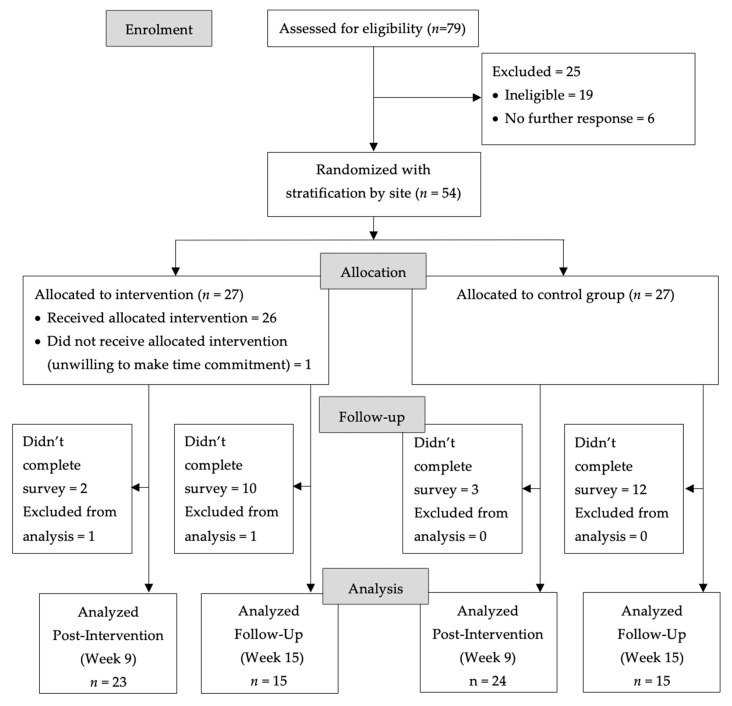
Coping with Cancer in the Kitchen trial flow diagram.

The Consolidated Standards of Reporting Trials (CONSORT) flowchart for the trial is shown in Figure 1. A total of 54 adult cancer survivors were randomly assigned to intervention (*n* = 27) and control groups (*n* = 27). The majority (76%) of study participants learned about CCK from CSC-Los Angeles and YMCA staff; 9% from their oncologist; and 4% from other care providers. There was only 1 drop-out in the intervention arm (4%) who declined participation after randomization but before the CCK program commenced, indicating they could no longer commit to the duration of the program. The retention rate was 91% at 9 weeks and 58% at 15 weeks. There were no statistically significant differences between those who completed the online 15-week follow-up survey and those who did not with respect to sociodemographic variables, disease characteristics, and baseline levels of primary and secondary dependent variables. One participant in the intervention arm was excluded from the analysis due to an ineligibility discovered after completion of the program. Attendance rates ranged from 100% for the first session to 84% for sessions 3 and 8. 

Characteristics for the total sample and by treatment group are shown in Table 2. Study participants were, on average, 61 years of age and primarily female breast cancer survivors with a college degree. The sample was 77% non-Hispanic white and 23% Hispanic or non-white race. Approximately half of the participants were married or living as married; 52% resided in suburban regions and 40% in urban areas. Most study subjects had overweight or obesity (65%), and less than half (45%) indicated they ate enough plant-based foods like fruits, vegetables, whole grains and beans in the past month (“most of the time” or “all of the time”). The CCK intervention group and printed materials control group were similar with respect to sociodemographic and disease characteristics.

Despite the randomization of participants, there were notable imbalances, though not statistically significant mean differences, between study groups in baseline levels of primary and secondary outcome measures (Table 3). Participants in the control group, on average, entered the study with higher confidence preparing a variety of plant-based foods and skills to practice a plant-based diet, and they also reported better quality of life, lower psychological distress, and less fatigue. Thus, we adjusted for baseline levels in regression analyses.

**Figure 2:**
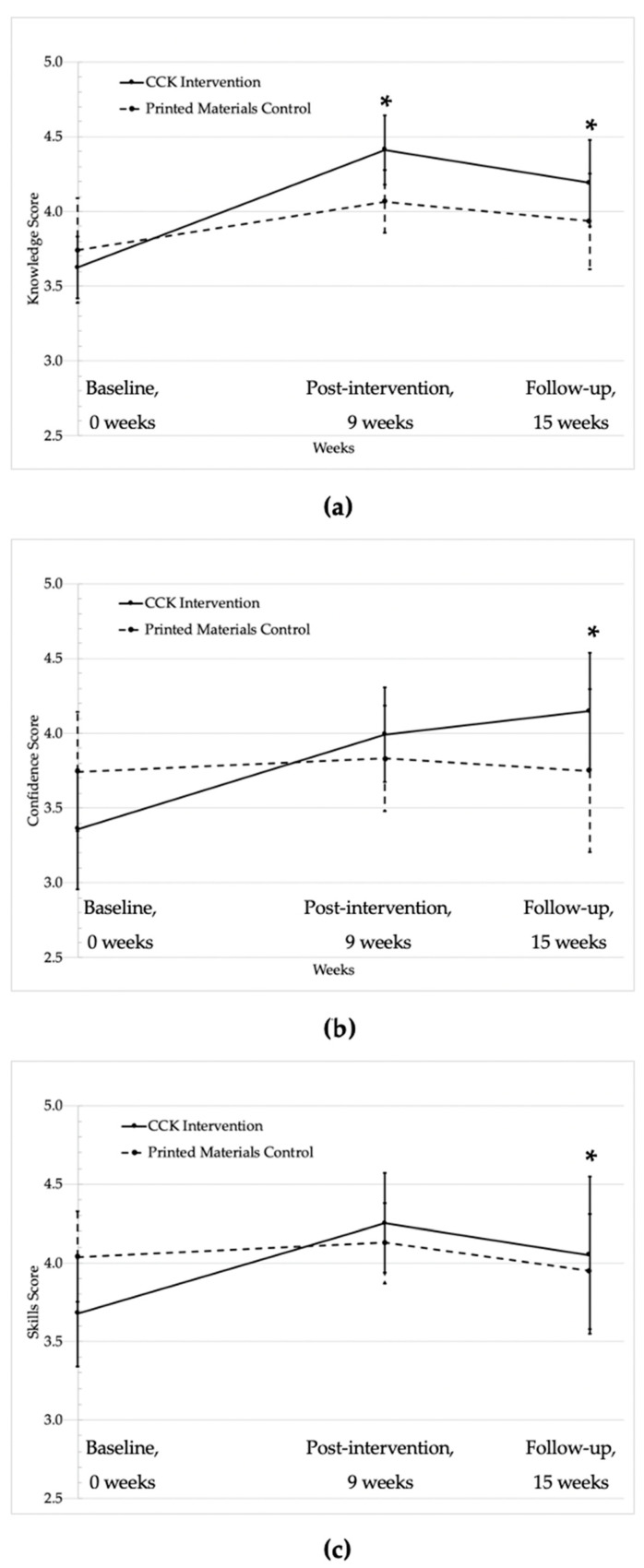
Change in (**a**) knowledge about a plant-based diet, (**b**) confidence preparing a variety of plant foods in a tasty way, and (**c**) skills to practice a plant-based diet for the Coping with Cancer in the Kitchen intervention group and the printed materials control group. * *p* < 0.05, difference between intervention and control groups adjusting for baseline level and study site. CCK: Coping with Cancer in the Kitchen.

Knowledge about a plant-based diet significantly increased in the intervention arm (in-person CCK program) compared to the control arm (printed materials); this increase was sustained at 15 weeks post-intervention (Figure 2a). Confidence in preparing plant-based foods significantly increased at 15 weeks (Figure 2b) as did level of skills to practice a plant-based diet (Figure 2c). Perceived barriers to eating F&V decreased in the CCK intervention group and increased in the control group, and the adjusted difference between intervention and control groups was statistically significant (−0.37; 95% confidence interval (CI) −0.64, −0.10; see Table 3). In addition, there was a larger decrease in perceived barriers to consuming whole grains in the intervention group compared to the control group (−0.3 v −0.1), but the treatment effect did not reach statistical significance (−0.24; 95% CI −0.56, 0.07).

Participants began the study consuming, on average, 2.78 and 2.64 cup equivalents (5.6 and 5.3 servings) for F&V per day in the intervention and control groups, respectively. Intake of whole grains was approximately 1.3 and 1.4 servings per day, respectively. The between-arm differences in intake of F&V or whole grains consumption were not statistically significant. Further, intake increased in both groups over time with adjusted differences between groups post-intervention of 0.17 cup equivalents (95% CI −0.13, 0.47), or 0.34 servings, per day for F&V among participants in the intervention group compared with those in the control group, and 0.06 ounce equivalents (95% CI −0.18, 0.30), or 0.11 servings, in whole grains. The CCK intervention group also had significantly lower daily servings of processed meat in comparison to the control group at 9 and 15 weeks. In the intervention group, intake of processed meat was 0.14 times per day at baseline, which is equivalent to approximately one (0.14 × 7 = 0.98) time per week, and it decreased to approximately one (0.04 × 30 = 1.2) time in the past month.

No statistically significant differences between CCK intervention and control groups were observed in self-reported assessments of quality of life. Nonetheless, the baseline to 9-week change trended in a positive direction for general quality of life (+1.0 v +0.2; FACT-G7), psychological distress (−0.4 v +0.5; PHQ-4), and fatigue (−2.1 v −0.8; 11-point Likert). Similarly, the level of social support and perceived control over the course of cancer were relatively stable in both arms, and the results suggested a trend for increase at 15 weeks in the CCK intervention group.

**Figure 3:**
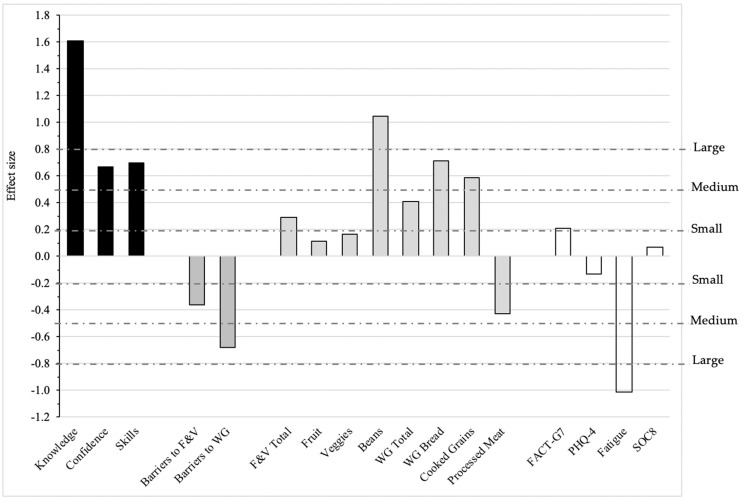
Effect size for the baseline to 9-week change in the Coping with Cancer in the Kitchen intervention group, as measured by the Effect Size statistic. The dotted lines correspond to small (0.2), medium (0.5), and large (0.8) effect sizes. WG: whole grain; F&V: fruit & vegetable; FACT-G7: a rapid version of the Functional Assessment of Cancer Therapy-General; PHQ-4: 4-item Patient Health Questionnaire for Depression and Anxiety; SOC8: NIH Toolbox^®^ Emotional Support Fixed Form Age 18+ v 2.0, Short Form.

The magnitude of effect sizes for the changes between baseline and 9 weeks (post-intervention) in the CCK intervention group, as measured by the *ES* statistic, are graphically presented in Figure 3. The effect sizes varied across categories of outcomes with large or nearly large effect sizes for outcomes measuring knowledge, confidence and skills. Barriers to consuming F&V and whole grains showed medium reductions. The effect sizes were in the range of small to medium for total F&V, whole grain and processed meat intake but were greater (with medium to large changes) for specific components of those dietary factors including beans and legumes, whole grain bread, and cooked whole grains (like quinoa). We detected small changes in quality of life measures. The decrease in fatigue was nonetheless large. We also considered using Cohen’s *d* as a measure of effect size, which is the 9-week difference between the CCK intervention and control groups divided by the pooled standard deviation, which is common when comparing two independent groups. However, baseline differences between the CCK intervention and control groups were large relative to the variability, or standard deviation, of the factor of interest, and, therefore, may have underestimated the true treatment effect.

## 4. Discussion

This trial investigated the effectiveness of CCK, a multidisciplinary behavioral intervention incorporating both nutrition education and psychosocial support, in modulating several motivational, action, and environmental mediators for implementing a healthy plant-based diet and for improving quality of life among cancer survivors. Previously published interventions have shown that motivation, goal setting, action planning, social support, and instruction regarding how to perform desired behaviors are key elements in successfully promoting behavioral changes whereas self-monitoring is often less effective in doing so [43]. CCK reflects those concepts in its curriculum, and the results from this randomized controlled trial favored in-person delivery of CCK over receipt of CCK printed material only.

### 4.1. Primary Outcomes (Knowledge, Cooking Confidence, Skills) and Perceived Barriers 

In-person delivery of the CCK program resulted in significant increases in knowledge, cooking confidence, and skills in adopting a plant-based diet over 9 and 15 weeks compared to the control group that received written CCK materials. Participants who attended CCK in person also reported a greater reduction in perceived barriers to the consumption of F&V and whole grains compared to the control group. As the literature indicates, people with higher perceived barriers tend to have poorer diets and are less likely to engage in behavioral changes, even when they are aware of the benefits of lifestyle changes [20,43,44]. Lack of access to accurate nutrition information, disbelief in diets and their relationship to cancer outcomes, low reinforcement from friends and family, and unfamiliarity with certain plant-based foods are commonly cited reasons for people not taking actions [20,44]. In particular, limited knowledge and skills in selecting and cooking healthy foods often demotivate cancer survivors from making dietary or lifestyle changes [44,45]. CCK addresses those concerns and obstacles by providing evidence-based nutrition education tailored to cancer survivors and delivered by registered dietitians. Moreover, CCK sessions included facilitator-led group discussions to enquire and consider approaches to reduce barriers to preparing and consuming a plant-based diet specifically for cancer survivors, and they contained weekly thematic cooking demonstrations using evidence-based AICR’s dietary recommendations and inviting participants for recipe tastings.

Facilitated group discussion, access to trained facilitators, and experiential culinary support may contribute substantially to the observed difference between the intervention and control arms. Participants in the CCK intervention group were able to observe thematic, plant-based foods and recipes being prepared, ask questions, receive verbal information in real time about the health benefits of the ingredients in CCK recipes, interact with group members during the recipe demonstrations, and were encouraged to taste new foods. Fredericks et al. state that nutritional education with experiential features provides further drivers for behavioral change including collaboration, peer support, and palate development [46]. Though telephone and web/app-based interventions can be more accessible to a wider audience, especially in remote areas, they rely heavily on self-monitoring and often face challenges in retaining participants [47]. Conversely, CCK’s in-person classes achieved high attendance (≥84%), which is comparable to other effective nutrition education programs targeting cancer survivors, such as Cocinar Para Tu Salud, a 12-week nutrition education program, and the Home Vegetable Gardening Interventions [48,49,50]. The study showed the benefit of in-person implementation over provision of printed materials only. However, given the current COVID-19 pandemic, future research could investigate virtual implementation of CCK using an online platform when in-person gatherings are prohibited, not possible or not preferred. The CCK program is available to survivors of all types of cancer. Its broad relevance increases the efficiency of delivery, the adaptability to local communities, and the scalability regionally and nationally.

The demonstrated enhancements in knowledge, skills, and confidence in practicing a plant-based diet at the end of the program, which continued their upward trend even at 15 weeks follow-up, implied, though not directly measured, the CCK effect on improving self-efficacy, which likely led to a higher level of patient empowerment [51,52]. Self-efficacy is a critical indicator of patient empowerment and a key construct of the Social Cognitive Theory and Transtheoretical Model used to guide the design and implementation of the CCK program for health behavior change [26,28,52]. Low self-efficacy (i.e., low confidence in one’s ability to execute a course of action) is an important barrier impeding behavioral change among cancer survivors [53,54]. Further, practicing and experiencing are among the most important sources of self-efficacy [26]. We indeed observed that CCK’s positive effect on self-efficacy was larger in the 15th week follow-up survey than immediately post-intervention (9 weeks) as participants had had more time to practice a plant-based diet by the 15th week. Higher self-efficacy has been indicated in studies to associate with higher probability of achieving and maintaining healthy behavioral goals and overall higher quality of life [55,56,57]. Further, empowerment also positively correlates with healthier behaviors and better decisions as well as health and clinical outcomes including improved disease management behaviors, use of health services, and health status [58,59]. 

### 4.2. Dietary Intake

The effects of CCK on total F&V and total whole grain intake were not statistically significant, but the observed net gain of 0.17 cup equivalents, or 0.34 servings, in daily F&V intake was similar to other studies of nutrition interventions designed to increase adult F&V intake [21,60]. A systematic review of the literature documented increases of 0.2 to 0.6 servings of F&V, and when targeting smaller focused communities, increases of 0.7 to 1.4 were observed [60]. The CCK participants had high baseline dietary intake of F&V at nearly 3 cups per day (exceeding the minimum intake recommended by AICR guidelines). Increasing people’s intake of nutrients or foods when the baseline intake is already sufficient is expected to be challenging [21] and might have contributed to our study not observing significant post-interventional dietary changes. As hypothesized, we demonstrated a significant reduction in the consumption of processed meats in the CCK intervention group and observed medium to large effect sizes in specific components of total whole grain intake (e.g., cooked grains and bread). These changes, coupled with significant increases in mediators of behavior change (knowledge, confidence and skills) suggests that with longer follow-up, participants are likely to continue making important changes in adopting a plant-based diet. Additional investigation in diverse populations and communities whose adherence to the recommended dietary guidelines is low is warranted.

### 4.3. Quality of Life and Other Psychosocial Measures 

There was modest impact on measurements of quality of life (QoL) in the current study, and effect sizes were generally small. Though not statistically significant, findings were within the range of effect sizes reported in prior research of interventions for cancer patients [61,62]. The data suggested positive trends in QoL, reduced fatigue and lower psychological distress at 9 weeks, though these trends did not reach statistical significance and were not sustained by the 15th week. Our limited sample size could be one of the major reasons for not finding statistical significance despite the observed positive trends. Likewise, a randomized controlled trial conducted by Uster and colleagues reported similar non-significant improvements in QoL among palliative cancer patients after nutrition and physical exercise interventions [63]. 

Living with cancer is undoubtedly stressful and associated with reduced QoL. Previous research demonstrated as many as 40% of cancer patients have clinically significant psychiatric comorbidities [64,65,66] and one in two reported significant distress [67]. Chronic stress is linked to several biobehavioral mechanisms related to the development of depressive symptoms and poorer cancer prognosis that may discourage people from making positive changes in their lifestyle; these factors may contribute to a vicious cycle of persistent emotional distress and accelerated physical deterioration [68]. Furthermore, it has been indicated that up to 60% of cancer survivors have never received psychosocial support due to limited access to such programs, suggesting the existence of unaddressed needs in this population [69]. We chose to measure health-related QoL using the FACT-G7 due to its brevity, validity, and reliability for use in cancer patients [37]. The Yanez (2013) study reported a mean score for the FACT-G7 of 18.0 among cancer patients and 19.5 in a general population sample [37]. In the current study, we reported an average baseline score of 17.1 among participants in the CCK intervention group with a one-point increase at 9 weeks post-intervention. The CCK intervention has the potential to close the gap between cancer survivors and the general population in health-related quality of life and mental health directly through learning coping strategies to reduce stress and indirectly through improving diet quality [70]. Furthermore, in a naturalistic study, Giese-Davis et al. showed that CSC therapist-led support groups provided an experience in which the development of a new attitude was valued [71].

### 4.4. Limitations

This trial was small, and a substantial proportion of participants did not complete the online follow-up survey at 15 weeks. The low rate of response to completing the 15-week survey may in part be attributed to the online platform, which was a departure from the pen-and-paper format of the previous surveys. Retention rates may have been higher if we had disseminated the questionnaire in the same format as the previous and additionally provided an incentive upon completion of the survey, either financial or educational by disseminating preliminary study findings to interested participants.

Limitations in our dietary measurement methods may also have prevented us from fully uncovering the true effects of CCK on dietary intake. The use of 24-h recall, food records, and objective biomarkers may be more sensitive to changes and warranted in future evaluations of CCK, which would allow us to quantify more nuanced changes in dietary intakes, such as replacing processed grains with whole grains rather than an overall increase in whole grains. Furthermore, follow-up was short. We recognize that behavioral change and changes in quality of life may take longer than 8–15 weeks. We hypothesized that participation in the CCK intervention would indirectly influence health-related behaviors through self-efficacy mediators. With more time to practice acquired skills, intake of plant-based foods may continue to increase in the CCK intervention arm with longer follow-up.

Another limitation of the study was that clinical outcomes were only measured through patient-report. We did not include body mass index or percent body fat as outcomes since the CCK program was not specifically designed to be a weight loss program. While adopting a healthier diet can result in weight loss or changes to body composition, the goals of cancer survivors can be varied with some aiming to increase body weight and others to lose or maintain weight. As the program is more widely disseminated and administered in academic and clinical settings, there may be increased feasibility and interest in measuring pro- and anti-inflammatory biomarkers (e.g., C-reactive protein, Interleukins 3, 6, 8, and 10, and Tumor Necrosis Factor-α) and plasma concentration of antioxidants. 

Other limitations included self-selected samples of participants who are predominantly female, White, fairly educated, and a substantial proportion of individuals with breast cancer. These limitations impact the study results’ generalizability to a more diverse population. However, while this sample is not representative of all cancer patients and survivors across the US [72], it is representative of those who tend to seek nutrition education as well as social and emotional support in their community. Future work is required to understand the impact that CCK has on participants’ long-term behavior changes, and to evaluate its applicability and cultural sensitivity among other diverse samples and settings. 

## 5. Conclusions

Participation in the in-person CCK intervention led to improvements in nutrition and food-related knowledge and skills as well as confidence in adopting a plant-based diet among cancer survivors. As an evidence-based, experiential nutrition education program that embeds psychosocial health elements, CCK can serve as the standard for a high-quality community-based survivorship program. Further studies and larger-scale implementation of CCK in more diverse populations are needed to further understand its effect on health-related behaviors and wellbeing. Promoting lifestyle behavioral change through programs like CCK has potential and value to improve cancer survivors’ nutritional status and quality of life. 

## Figures and Tables

**Table 1:** Description of intervention components and outcome measures used in a randomized controlled trial of Coping with Cancer in the Kitchen, a Nutrition Education Program for Cancer Survivors

Measure/Component	Description
Intervention Components	
Eight, in-person, 90-min group meetings convened weekly at community-based organizational facilities	
(1) Nutrition Education	Registered dietitians educated participants using slide presentations about the American Institute for Cancer Research’s (AICR’s) Recommendations for Cancer Prevention, “New American Plate^®^”, and “Foods That Fight Cancer (FTFC)™” using 8 modules. Module 1: AICR Recommendations for Cancer Prevention Module 2: AICR’s New American Plate and One-Pot Meals Module 3: Beans and Whole Grains Module 4: Breakfast and Snacks Module 5: Comfort Foods Module 6: Veggies Module 7: Building a FTFC Kitchen Module 8: Sharing and Caring Potluck
(2) Structured Group Learning and Support	Licensed social workers provided support through a structured and empowering group learning environment to address the complex, important (and, unfortunately, often rarely openly discussed) psychosocial barriers to nutrition behavior change in the context of cancer prevention, treatment, and survivorship.
(3) Cooking Demonstration	Culinary experts demonstrated convenient, easy, and tasty ways to prepare and cook FTFC and invited recipe tasting.
Sharing and Caring Potluck	The last in-person meeting of the program included a time to review the overall experience and engage in discussion. It was intended to explore milestones achieved, recognize precipitous moments of comprehension, connect to feelings related to the program ending, identify ongoing obstacles and/or challenges, identify changes and successes along the way, share ideas and hopes for continued success, and discuss take-aways from the group experience.
Recipe Cards	Each week participants received 2–3 printed recipe cards for foods exhibited and tasted during the culinary demonstration. Examples of recipes included Quinoa Salad, Everyday Green Smoothie, Southwestern Bean Salad, Buckwheat Cocoa-Chip Overnight Oats, Chili, and Whole Wheat Greek Pasta Salad.
Workbook	Pocket folders included written materials about dietary choices and recipe cards.
S.M.A.R.T Goal-Setting Worksheets	Each week participants completed a one-page worksheet that prompted them to identify one to three specific, measurable, actionable, relevant and time-bound goals that were revisited at the next session.
**Control Group Components**	
Printed Educational Materials	Participants in the control group received seven comprehensive summaries from Coping with Cancer in the Kitchen weekly module content and 14 recipe cards (two from each of the 7 weeks of culinary demonstrations). These were mailed to participants in one package upon completion of the baseline survey.
**Pre–Post Outcome Measures**	
Knowledge about a Plant-Based Diet	Participants rated their agreement (1 = *Strongly disagree*; 5 = *Strongly agree*) with six custom items developed by the research team, e.g., “I understand the benefits of consuming whole grains versus processed grains”. A composite score was calculated as the average of the 6 ratings (range 1–5; Cronbach’s alpha = 0.80).
Confidence Preparing a Variety of Plant Foods	Participants indicated “How sure are you that you could prepare the foods listed below in a tasty way?” (1 = *Very unsure*; 5 = *Very sure*). The 14-item scale included 4 whole grains; 4 beans, seeds and legumes; 3 green leafy vegetables; and 3 mixed foods, e.g., healthy one-pot meals. A composite score was calculated as the average of the 14 items (range 1–5; Cronbach’s alpha = 0.75).
Skills to Practice a Plant-Based Diet	Participants rated their agreement (1 = *Strongly disagree*; 5 = *Strongly agree*) with five custom items developed by the research team, e.g., “I am confident that I can create a kitchen environment that makes it easier to store, prepare, and consume fruits, vegetables, whole grains, and beans.”; the average of the five ratings was calculated to create a skills composite score (range 1–5; Cronbach’s alpha = 0.88).
Barriers to Eating More Fruits and Vegetables and Whole Grains	We adapted items from an existing barriers instrument [33,34] to measure perceived barriers to eating more fruits and vegetables (F&V) (average score of 15 items; Cronbach’s alpha = 0.89) and whole grains (average score of 14 items; Cronbach’s alpha = 0.83). Participants were asked the general question, “Listed below are some common reasons why people don’t eat more servings of *vegetables and fruits* each day. Indicate whether or not this is a reason for you by marking how much you agree or disagree.” (1 = *Strongly disagree*; 5 = *Strongly agree*). In addition, using the same list of possible reasons (excluding *spoil too quickly*), participants indicated whether it was a common reason they did not eat more servings of *whole grains*. Example reasons included *take too much time to prepare; my family doesn’t like them; hard to find a variety of good ones*.
Dietary Intake [Dietary Screener Questionnaire (DSQ) in the NHANES 2009-10] https://epi.grants.cancer.gov/nhanes/dietscreen/	A 26-item dietary screener developed by the National Cancer Institute [35], which we shortened to include 17 questions that ask about the frequency of intake in the past month of F&V, whole grains, and processed and red meats. Scoring algorithms convert screener responses to estimates of daily intake of cup equivalents of F&V, including legumes and excluding French fries, and whole grains (ounce equivalents). Frequency responses to the processed meat question is converted to times per day. The DSQ provides a less burdensome alternative to 24-h recall when interest is in a limited set of dietary factors. We piloted use of the DSQ during the 2017 single-arm pilot study; intake was comparable to those participating in the National Health and Nutrition Examination Survey 2009–2010 [35].
General Quality of Life [a rapid version of the Functional Assessment of Cancer Therapy-General (FACT-G7)]	The Functional Assessment of Cancer Therapy-General (FACT-G) questionnaire is a general quality of life instrument that can be used to assess top-rated symptoms and concerns in cancer patients [36]. The FACT-G7 is a brief 7-item adaptation [37]. Internal consistency and reliability in the present study was good (Cronbach’s alpha = 0.80).
Psychological Distress [4-item Patient Health Questionnaire for Depression and Anxiety (PHQ-4)]	The PHQ-4 is a brief 4-item validated screening scale for measuring core symptoms and signs of depression and anxiety [38].
Fatigue	Participants were asked to rate their level of fatigue on the average in the last week [0 = *Not at all fatigued*; 10 = *Fatigued as I could be*]. This item comes from the Fatigue Symptom Inventory that assesses the frequency and severity of fatigue and its perceived interference [39].
Emotional Support [NIH Toolbox^®^ Emotional Support Fixed Form Age 18+ v 2.0, Short Form (SOC8)]	Participants completed the SOC8 which measures emotional support, or the perceived availability of someone to provide empathy or advice in times of need [40]. Higher scores represent more emotional support. Scores are converted to standardized *T* scores (mean = 50, standard deviation = 10); normative reference groups are the US general population.
Perceived Control over Course of Cancer	Participants were asked, “To what extent do you feel you have control over the course of your cancer (that is, whether your cancer will come back, get worse, or you will develop a different type of cancer)?” (0 = *No control at all*; 4 = *Complete control*).

**Table 2:** Sociodemographic and disease characteristics of cancer survivors participating in a randomized study of Coping with Cancer in the Kitchen

		Total Sample (N = 53)			Coping with Cancer in the Kitchen Intervention (*n* = 26)			Printed Materials Control (*n* = 27)		*p*-Value ^a^
Factor	No. of Participants		%	No. of Participants		%	No. of Participants		%	
**Sociodemographic Characteristics**										
Age, years										0.25
Mean		61.2			59.5			62.8		
SD		10.5			9.7			11.1		
Range		37–80			37–74			41–80		
Gender										0.99
Female	49		92	24		92	25		93	
Male	4		8	2		8	2		7	
Race										0.43
Non-Hispanic white	41		77	18		69	23		85	
Non-Hispanic black	4		8	2		8	2		7	
Non-Hispanic, other	6		11	4		15	2		7	
Hispanic or Latino	2		4	2		8	0		0	
Education										0.30
Did not graduate college	14		26	6		23	8		30	
College graduate	16		30	6		23	10		37	
Postgraduate	23		43	14		54	9		33	
Geographic region										0.53
Urban	21		40	11		44	10		37	
Suburban	27		52	11		44	16		59	
Rural	3		6	2		8	1		4	
Marital status										0.43
Married or living as married	27		52	15		60	12		44	
Single (never married)	13		25	6		24	7		26	
Divorced or separated	9		17	4		16	5		19	
Widowed	3		6	0		0	3		11	
**Disease Characteristics**										
Time since cancer diagnosis										0.57
Mean		4.9			5.4			4.5		
SD		5.9			6.8			5.0		
Range		<1 to 27			<1 to 27			<1 to 21		
Primary cancer diagnosis										0.18
Breast	25		47	9		35	16		59	
Metastatic breast	6		11	5		19	1		4	
Blood	5		9	2		8	3		11	
Female reproductive	4		8	3		12	1		4	
Multiple cancers specified	6		11	2		8	4		15	
Other	7		13	5		19	2		7	
Ever received chemotherapy										
Yes	29		56	14		56	15		56	0.97
No	23		44	11		44	12		44	
**Health Status**										
Perceived health										0.87
Excellent	0		0	0		0	0		0	
Very good	15		31	8		33	7		28	
Good	23		47	11		46	12		48	
Fair	10		20	4		17	6		24	
Poor	1		2	1		4	0		0	
Body Mass Index, kg/m^2^										0.84
Underweight (<18.5)	2		4	1		4	1		4	
Normal (18.5–24.9)	17		32	10		38	7		26	
Overweight (25.0–29.9)	13		25	6		23	7		26	
Obese (>30)	21		40	9		35	12		44	
In the last month, ate enough plant-based foods										0.73
None of the time	1		2	1		4	0		0	
A little of the time	7		14	4		17	3		12	
Some of the time	19		39	8		33	11		44	
Most of the time	16		33	9		38	7		28	
All of the time	6		12	2		8	4		16	

^a^ Two-sample *t*-tests for continuous variables and Fisher’s exact test for categorical variables. SD: standard deviation.

**Table 3:** Baseline, post-intervention (9-week), and follow-up (15-week) self-reported outcomes observed for the Coping with Cancer in the Kitchen intervention group and the printed materials control

		Coping with Cancer in the Kitchen Intervention			Printed Materials Control		Adjusted Difference (95% CI) between Intervention and Control Groups ^b^	
Factor	Baseline (*n* = 24)	Post-Intervention (*n* = 23)	15-Week Follow-Up (*n* = 15)	Baseline (*n* = 25)	Post-Intervention (*n* = 24)	15-Week Follow-Up (*n* = 15)	Post-Intervention	15-Week Follow-Up
**Primary Outcomes**								
Knowledge about a Plant-Based Diet							0.36 * (0.06, 0.67)	0.54 * (0.11, 0.98)
Mean	3.6	4.4	4.2	3.7	4.1	3.9		
SD	0.5	0.5	0.5	0.8	0.5	0.6		
Within-arm mean difference		+0.8 ^c,^*	+0.6 ^d,^*		+0.4 ^e^	+0.2 ^f^		
Confidence Preparing a Variety of Plant Foods							0.36 (−0.02, 0.74)	0.83 * (0.23, 1.42)
Mean	3.4	4.0	4.1	3.7	3.8	3.7		
SD	0.9	0.8	1.0	1.0	0.8	0.7		
Within-arm mean difference		+0.6 *	+0.7 *		+0.1	0		
Skills to Practice a Plant-Based Diet							0.28 (−0.09, 0.64)	0.65 * (0.16, 1.14)
Mean	3.7	4.3	4.1	4.0	4.1	3.9		
SD	0.8	0.7	0.9	0.7	0.	0.7		
Within-arm mean difference		+0.6 *	+0.4 *		+0.1	−0.1		
**Perceived Barriers**								
Perceived Barriers to Eating More Fruits and Vegetables							−0.37 * (−0.64, −0.10)	NA
Mean	2.6	2.4	--	2.4	2.5	--		
SD	0.6	0.7	--	0.8	0.8	--		
Within-arm mean difference		−0.2 *	--		+0.1	--		
Perceived Barriers to Eating More Whole Grains							−0.24 (−0.56, 0.07)	NA
Mean	2.6	2.3	--	2.6	2.5	--		
SD	0.5	0.7	--	0.6	0.7	--		
Within-arm mean difference		−0.3 *	--		−0.1	--		
**Dietary Intake**								
Total Fruit and Vegetable, cup equivalents per day ^g^							0.17 (−0.13, 0.47)	−0.19 (−0.55, 0.17)
Mean	2.78	2.99	2.86	2.64	2.70	2.76		
SD	0.73	0.85	0.84	0.76	0.76	0.88		
Within-arm mean difference		+0.21	+0.08		+0.06	+0.12		
Whole Grains Total, ounce equivalents per day ^h^							0.06 (−0.18, 0.30)	0.01 (−0.24, 0.27)
Mean	0.71	0.83	0.82	0.78	0.82	0.89		
SD	0.30	0.46	0.36	0.49	0.39	0.43		
Within-arm mean difference		+0.12	+0.11		+0.04	+0.11		
Processed Meat, times per day							−0.08 * (−0.15, −0.02)	−0.09 * (−0.18, −0.01)
Mean	0.14	0.04	0.04	0.08	0.10	0.11		
SD	0.23	0.05	0.05	0.15	0.18	0.17		
Range		−0.10 *	−0.10 *		+0.02	+0.03		
**Quality of Life**								
General Quality of Life, FACT-G7 Score							0.63 (−1.45, 2.71)	−0.09 (−3.54, 3.36)
Mean	17.1	18.1	17.1	17.8	18.0	18.4		
SD	5.1	5.0	6.1	5.9	5.7	4.2		
Within-arm mean difference		+1.0	0		+0.2	+0.6		
Psychological Distress, PHQ-4							-0.83 (−2.13, 0.46)	−0.66 (−2.92, 1.60)
Mean	3.25	2.87	3.53	2.67	3.17	2.73		
SD	2.83	2.82	3.40	2.41	3.51	3.06		
Within-arm mean difference		−0.38	+0.28		+0.50	+0.06		
Fatigue, range 0–10							−0.76 (−2.09, 0.57)	−0.22 (−1.59, 1.14)
Mean	5.9	3.8	4.1	5.1	4.3	3.7		
SD	2.1	2.4	2.3	2.3	2.6	2.0		
Within-arm mean difference		−2.1 *	−1.8 *		−0.8	−1.4		
Emotional Support, SOC8 T-score							0.17 (−3.23, 3.57)	1.67 (−3.80, 7.13)
Mean	44.1	44.8	47.7	45.6	46.1	46.9		
SD	9.9	8.4	9.8	11.2	11.2	12.6		
Within-arm mean difference		+0.7	+3.6		+0.5	+1.3		
Perceived Control Over Course of Cancer, range 0–4							−0.18 (−0.63, 0.27)	0.34 (−0.20, 0.88)
Mean	1.4	1.5	1.9	1.1	1.4	1.0		
SD	1.1	0.9	0.8	1.2	1.0	1.1		
Within-arm mean difference		+0.1	+0.5		+0.3 *	−0.1		

* *p* < 0.05, paired t-test. * Bolded, *p* < 0.05, from linear regression analysis. ^b^ From linear regression analysis adjusting for baseline level and study site. Intent-to-treat analysis that used last observation carried forward for missing data did not appreciably change results or affect significance. ^c^ Difference computed from columns 3 and 2 (post-intervention minus baseline values) within the CCK group. ^d^ Difference computed from columns 4 and 2 (follow-up minus baseline values) within the CCK group. ^e^ Difference computed from columns 6 and 5 (post-intervention minus baseline values) within the control group. ^f^ Difference computed from columns 7 and 5 (follow-up minus baseline values) within the control group. ^g^ ½ cup equivalent fruit and vegetable = 1 daily serving. ^h^ A total of ~0.56 oz equivalent whole grains = 1 daily serving. SD: standard deviation; CI: confidence interval; NA: not applicable; FACT-G7: a rapid version of the Functional Assessment of Cancer Therapy-General; PHQ-4: 4-item Patient Health Questionnaire for Depression and Anxiety; SOC8: NIH Toolbox^®^ Emotional Support Fixed Form Age 18+ v 2.0, Short Form
